# Rotavirus vaccine and health-care utilization for rotavirus gastroenteritis in Tsu City, Japan

**DOI:** 10.5365/WPSAR.2016.7.3.005

**Published:** 2016-11-14

**Authors:** Kazutoyo Asada, Hajime Kamiya, Shigeru Suga, Mizuho Nagao, Ryoji Ichimi, Takao Fujisawa, Masakazu Umemoto, Takaaki Tanaka, Hiroaki Ito, Shigeki Tanaka, Masaru Ido, Koki Taniguchi, Toshiaki Ihara, Takashi Nakano

**Affiliations:** a Department of Pediatrics, National Hospital Organization Mie Hospital, Tsu, Japan.; b Infectious Disease Surveillance Center, National Institute of Infectious Diseases, Shinjuku, Japan.; c Department of Pediatrics/Neonatology, Ise Red Cross Hospital, Ise, Japan.; d Umemoto Children’s Clinic, Tsu, Japan.; e Department of Pediatrics, Kawasaki Medical School, Kurashiki, Japan.; f Department of Pediatrics, Kameda Medical Center, Kamogawa, Japan.; g Department of Pediatrics, Mie-Chuo Medical Center, Tsu, Japan.; h Department of Virology and Parasitology, Fujita Health University School of Medicine, Toyoake, Japan.

## Abstract

**Background:**

Rotavirus vaccines were introduced in Japan in November 2011. We evaluated the subsequent reduction of the health-care burden of rotavirus gastroenteritis.

**Methods:**

We conducted active surveillance for rotavirus gastroenteritis among children under 5 years old before and after the vaccine introduction. We surveyed hospitalization rates for rotavirus gastroenteritis in children in Tsu City, Mie Prefecture, Japan, from 2007 to 2015 and surveyed the number of outpatient visits at a Tsu City clinic from 2010 to 2015. Stool samples were obtained for rotavirus testing and genotype investigation. We assessed rotavirus vaccine coverage for infants living in Tsu City.

**Results:**

In the pre-vaccine years (2007–2011), hospitalization rates for rotavirus gastroenteritis in children under 5 years old were 5.5, 4.3, 3.1 and 3.9 cases per 1000 person-years, respectively. In the post-vaccine years (2011–2015), the rates were 3.0, 3.5, 0.8 and 0.6 cases per 1000 person-years, respectively. The hospitalization rate decreased significantly in the 2013–2014 and 2014–2015 seasons compared to the average of the seasons before vaccine introduction (*P* < 0.0001). In one pre-vaccine year (2010–2011), the number of outpatient visits due to the rotavirus infection was 66. In the post-vaccine years (2011–2015), the numbers for each season was 23, 23, 7 and 5, respectively. The most dominant rotavirus genotype shifted from G3P[8] to G1P[8] and to G2P[4]. The coverage of one dose of rotavirus vaccine in Tsu City was 56.5% in 2014.

**Conclusion:**

After the vaccine introduction, the hospitalization rates and outpatient visits for rotavirus gastroenteritis greatly decreased.

## Introduction

In young children, the single most important cause of severe dehydrating diarrhoea is rotavirus infection. (*1*) Some patients need fluid therapy at the hospital for severe dehydration. Even in small numbers, death from rotavirus infection does occur in developed countries, including Japan. (*2*) Complications of rotavirus infection include seizure, prerenal or postrenal kidney failure and encephalitis/encephalopathy. (*3*-*5*) A study in Japan suggested rotavirus is the third leading pathogen of infections that proceed acute encephalopathy nationally after influenza virus and human herpesvirus-6. (*5*) Therefore, rotavirus vaccine would help reduce severe acute gastroenteritis and its complications.

In Japan, monovalent rotavirus vaccine (RV1) was introduced in November 2011 and pentavalent rotavirus vaccine (RV5) in July 2012. Currently, the rotavirus vaccine is not included in the National Immunization Programme in Japan, and the cost of vaccination including an administration fee is covered by parents and guardians. RV1 is administered at 2 and 4 months of age. RV5 is administered at 2, 3 and 4 months of age.

Previously, we studied the disease burden of rotavirus infection in children under 5 years old retrospectively in two cities (Tsu City, Ise City) from 2003 to 2007 in Mie Prefecture, Japan. (*6*) The annual hospitalization rate for rotavirus gastroenteritis in the two cities was estimated to be 3.8 and 4.9 per 1000 person-years, respectively.

Since then, we have been conducting active surveillance for rotavirus gastroenteritis hospitalization in children under 5 years old in three cities (Matsusaka City in addition to the two cities mentioned above) in Mie. (*7*) The annual hospitalization rate for rotavirus gastroenteritis in the three cities from 2007 to 2009 was estimated to be 2.8 to 4.7 per 1000 person-years.

In this study, we report monitored trends in the hospitalization rate and the number of outpatient visits due to rotavirus gastroenteritis, and prevalent rotavirus genotypes in Tsu City, Mie, Japan before and after the introduction of rotavirus vaccine.

## Methods

### Data source and case definition

We conducted active surveillance for rotavirus gastroenteritis among children under 5 years old in Tsu City, Mie, Japan before and after the vaccine introduction. In Japan, November to July is considered to be the rotavirus peak season and August to October is the rotavirus off-season. We defined one season as November of one year to October of the next year.

From November 2007 to October 2015, we surveyed hospitalization rates for rotavirus gastroenteritis in children under 5 years old. Two hospitals in Tsu City were included in this study because there are no other hospitals in the city that admit children with severe dehydration. In addition, we asked surrounding city hospitals to notify us if rotavirus acute gastroenteritis patients under 5 years old who reside in Tsu City were admitted to their hospitals.

From November 2010 to October 2015, we concurrently surveyed outpatient visits of children under 5 years old who were diagnosed with rotavirus gastroenteritis at one walk-in clinic in the same city. We selected this clinic in Tsu City because it has the most outpatient visits.

All patients under 5 years old who were hospitalized with a diagnosis of acute gastroenteritis were tested for rotavirus at the two hospitals. For patients from whom we were unable to collect stool samples at the time of admission, we attempted to collect samples during hospitalization. We did not use enema and rectal swab to collect samples. For the outpatient clinic, parents and guardians were asked to submit their child’s stool sample.

We used a commercially available enzyme immunoassay (Rota-Adeno Dry; Sekisui Medical Co., Tokyo, Japan) for rotavirus antigen detection in the stool specimens; the sensitivity and specificity of this test are approximately 94% and 99%, respectively, when compared with electron microscopy (data from package insert). Rapid inspection using this assay for rotavirus is broadly implemented in Japan. Positive cases by this testing were diagnosed as rotavirus gastroenteritis. Patients living outside Tsu City were excluded from this study.

### Genotype investigation

For rotavirus-positive stool samples, G and P genotypes were investigated. Stool suspension was prepared in Eagle’s minimum essential medium; rotavirus RNA was extracted for the determination of G and P types by nested reverse transcription polymerase chain reaction (RT–PCR) carried out in two steps, first and second amplifications, as described previously. (*8*, *9*) For G typing, the full-length VP7 gene was amplified using a pair of primers, 5′-GGCTTTAAAAGAGAGAATTTCCGTCTGG-3′ (T31) and 5′-GGTCACATCATACAATTCTAATCTAAG-3′ (T32), corresponding to the common 5′ and 3′ ends of the VP7 gene, respectively. In the second PCR amplification, the T32 primer was used along with G1, G2, G3, G4, G8 and G9 genotype-specific primers to identify G types. For P typing, a pair of primers, 5′-TGGCTTCGTTCATTTATAGACA-3′ and 5′-CTAAATGCTTTTGAATCATCCCA-3′, corresponding to the common sequences of the VP4 gene, including nucleotides 11 to 32 and 1072 to 1094, respectively, were used for the first amplification. A mixture of primers specific to each of the variable regions P[8], P[4], P[6] and P[9], along with a primer corresponding to nucleotides 11 to 32, were used for the second amplification. PCR products were electrophoresed in 1% agarose gels and stained with ethidium bromide.

### Estimation of rotavirus vaccine coverage

We defined the period from 2007 to 2011 as pre-vaccine years because the rotavirus vaccine was not commercially available until late November 2011; the period from 2012 to 2015 was defined as post-vaccine years. However, because the rotavirus vaccine is not routinely recommended in Japan, there is no official method to obtain the vaccine coverage rate for Tsu City. Thus, we estimated the rotavirus vaccine coverage rate using child health check-up data.

In Japan, all children are obliged to have periodic health check-ups by the government at 3 to 4, 18 and 36 months of age. We assessed rotavirus vaccine coverage at the 18-month check-up from January to March of 2014. We checked the immunization records of the mother–child handbook of these children to obtain the rotavirus vaccine coverage among children born in mid- to late 2012.

### Data analysis

We summarized the demographic characteristics of hospitalized cases and outpatient visits for rotavirus gastroenteritis using a standardized abstraction form. For hospitalizations, we calculated the annual incidence rate for each year using the total number of rotavirus-positive cases during the study period as the numerator and the population of those aged under 5 years as the denominator. We obtained population data from the statistics office in Mie every year for the number of children under 5 years old in the city.

We performed χ^2^ tests using the software GraphPad Prism version 6.0 (GraphPad Software Inc., San Diego, CA, USA). A *p*-value of less than 0.05 was considered statistically significant.

### Ethics

This study was approved by the Institutional Review Board of National Hospital Organization Mie Hospital.

## Results

### Trends in hospitalization for rotavirus gastroenteritis

**Table 1** and **Fig. 1a** summarized the yearly hospitalization rates for rotavirus gastroenteritis from 2007 to 2015. The average hospitalization rate in pre-vaccine years for children under 5 years old (2007–2011) was 4.2 cases per 1000 person-years (95% confidence interval, 3.7–4.8). The hospitalization rates in the post-vaccine years (2011–2012, 2012–2013, 2013–2014 and 2014–2015) were 3.0, 3.5, 0.8 and 0.6 cases per 1000 person-years, respectively. The hospitalization rate declined by 85.7% in 2014–2015 compared to the average of pre-vaccine years (0.6 and 4.2 cases per 1000 person-years, respectively). In the 2013–2014 and 2014–2015 seasons, the rate of hospitalizations was significantly lower compared with the seasons before vaccine introduction from 2007 to 2011 (*P* < 0.0001). There was no case admitted to surrounding city hospitals during the study period. No death or serious complication was observed during this study period.

**Table 1:** Hospitalization data for rotavirus gastroenteritis in Tsu City

-	Pre-vaccine years				Post-vaccine years			
	2007–2008	2008–2009	2009–2010	2010–2011	2011–2012	2012–2013	2013–2014	2014–2015
No. of hospitalizations	68	53	38	46	35	41	9	7
Tsu City population (< 5 years old)	12 270	12 339	12 279	11 755	11 775	11 794	11 687	11 598
Hospitalization rate (per 1 000 person-years)	5.5	4.3	3.1	3.9	3.0	3.5	0.8*	0.6*
95% confidence interval	4.4–7.0	3.3–5.6	2.3–4.2	2.9–5.2	2.1–4.1	2.6–4.7	0.4–1.5	0.3–1.2

* Statistically significant decrease compared to the average hospitalization rate before introduction of rotavirus vaccine (2007–2011).

**Fig. 1a:**
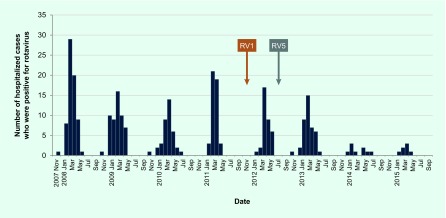
Number of hospitalizations for rotavirus gastroenteritis

Arrows indicate the times when the vaccines were introduced. RV1 is the monovalent rotavirus vaccine, and RV5 is the pentavalent rotavirus vaccine.

### Age distribution of hospitalizations

**Fig. 2:**
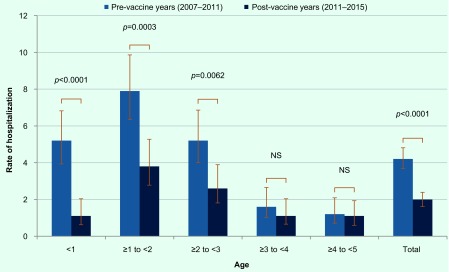
Age distribution of hospitalization rate for rotavirus gastroenteritis

**Fig. 2** shows hospitalization rates by age group. In the pre-vaccine years, 205 children were hospitalized for rotavirus gastroenteritis. Hospitalization rates per 1000 population were 5.2 among children aged under 1 year, 7.9 among children aged 1–2 years, 5.2 among children aged 2–3 years, 1.6 among children aged 3–4 years and 1.2 among children aged 4–5 years. In the post-vaccine years, 92 children were hospitalized. Hospitalization rates per 1000 population were 1.1 among children aged under 1 year, 3.8 among children aged 1–2 years, 2.6 among children aged 2–3 years, 1.1 among children aged 3–4 years and 1.1 among children aged 4–5 years. The hospitalization rates in the three age groups (under 1 year old, 1–2 years old and 2–3 years old) in the post-vaccine years decreased significantly compared with the pre-vaccine years (*P* < 0.0001, *P* = 0.0003 and *P* = 0.0062, respectively), while the hospitalization rates in the other age groups (3 years old or older) did not change significantly.

NS, not significant.

Bars indicate 95% confidence interval.

### Trends in outpatient rotavirus gastroenteritis cases

Outpatient visits were surveyed for just one season in the pre-vaccine years (2010–2011), in which there were 66 rotavirus gastroenteritis diagnosed cases. In the four post-vaccine seasons (2011–2012, 2012–2013, 2013–2014 and 2014–2015), there were 23, 23, 7 and 5 diagnosed rotavirus cases, respectively. A very sharp decrease in the number of rotavirus-positive cases was observed in the 2013–2014 season (**Fig. 1b**).

**Fig. 1b:**
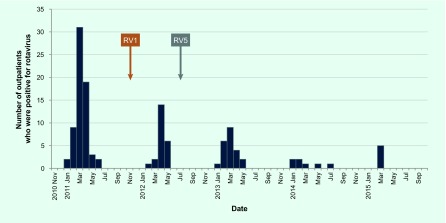
Number of outpatient visits for rotavirus gastroenteritis

Arrows indicate the times when the vaccines were introduced. RV1 is the monovalent rotavirus vaccine, and RV5 is the pentavalent rotavirus vaccine.

### Changes in genotypes

**Fig. 3:**
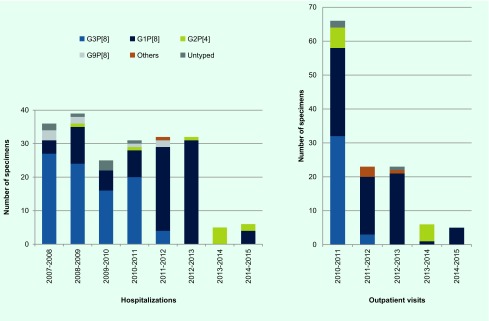
Changes of rotavirus genotypes of stools from hospitalized cases (left) and outpatient settings (right) in Tsu City, Japan

Of the 297 hospitalized patients, 206 (69.4%; 52.9–91.4%) were subjected to G and P typing using semi-nested PCR. Some stool samples were insufficient in quantity to investigate the genotype. From 2007 to 2011, the most dominant rotavirus genotype was G3P[8] (61.5–75.0%) followed by G1P[8] (11.1–28.2%) (**Fig. 3****)**. In 2011 to 2012 and 2012 to 2013, the most dominant rotavirus genotype was G1P[8] (78.1–96.9%). In 2013 to 2014, all five specimens tested had G2P[4]; in 2014 to 2015, G1P[8] (66.7%) was dominant from the six specimens tested.

Stool samples of all of the 123 outpatients were subjected to G and P typing (**Fig. 3**). In 2010 to 2011, the most dominant rotavirus genotype was G3P[8] (48.5%), and the second most dominant genotype was G1P[8] (39.4%). In 2011–2012 and 2012–2013, the most dominant rotavirus genotype was G1P[8] (73.9% and 91.3%, respectively). In 2013–2014, G2P[4] (83.3%) was dominant in the six specimens tested, and in 2014 to 2015, all five specimens tested had G1P[8].

### Estimated rotavirus vaccine coverage

Vaccination histories were collected at the 18-month check-ups from January to March of 2014. During that time, of 555 children who were required to have an 18-month check-up in the city, 543 visited health centres (98% compliance). The first dose of rotavirus vaccine had been administered to 56.5% of the children (307 out of 543; 251 received RV1 and 56 received RV5). The second dose of rotavirus vaccine had been administered to 54.9% of the children (298 out of 543; 243 children received RV1 and 55 received RV5). The third dose of RV5 had been administered to 9.6% children (52 out of 543). Of the 543 children, 44.8% completed the two-dose series of RV1, and 9.6% completed the three-dose series of RV5, giving the coverage of complete rotavirus vaccine series of 54.4%.

### Rotavirus gastroenteritis among vaccinated cases

Ten cases of rotavirus gastroenteritis were reported among vaccinated children, including four hospitalized cases and six outpatients (**Table 2**). All these cases were fully vaccinated with two doses of RV1 vaccine. G1P[8] was found in five cases and G2P[4] in four cases. Genotyping was not performed for one case due to insufficient specimen.

**Table 2:** Cases with vaccination history

Season	Age (month)	Sex	Inpatient or outpatient	Underlying condition	Vaccine type	Dose	Days from first dose to onset	Genotype
2011–2012	6	M	Inpatient	None	RV1	2	89	G1P[8]
2012–2013	9	M	Inpatient	None	RV1	2	221	G1P[8]
	10	F	Outpatient	None	RV1	2	233	G1P[8]
2013–2014	26	M	Inpatient	None	RV1	2	756	G2P[4]
	28	M	Outpatient	None	RV1	2	801	G2P[4]
	9	M		None	RV1	2	194	G2P[4]
	12	M		None	RV1	2	302	Untyped
2014–2015	21	F	Inpatient	None	RV1	2	569	G2P[4]
	34	M	Outpatient	None	RV1	2	972	G1P[8]
	34	M		None	RV1	2	993	G1P[8]

## Discussion

We actively surveyed both hospitalized and walk-in patients for laboratory-confirmed rotavirus acute gastroenteritis in Tsu City, Mie, Japan before and after the introduction of rotavirus vaccine. The average hospitalization rate in the pre-vaccine years was 4.2 cases per 1000 person-years, which is comparable to the reports from other developed countries in the pre-vaccine years: 2.7 cases per 1000 person-years in the United States of America, 3.7 to 13 cases per 1000 person-years in western Europe and 8.7 cases per 1000 person-years in Australia. (*10*-*15*) Hospitalization rates and outpatient visits for rotavirus gastroenteritis have greatly decreased after vaccine introduction in Tsu City. The hospitalization rate declined by 85.7% from 4.2 in pre-vaccine years to 0.6 cases per 1000 person-years in the 2014–2015 season. In other words, 42 hospitalizations were prevented among children under 5 years old in Tsu City, assuming the incidence without vaccination remained the same as baseline. If we extrapolate our results to a national population, assuming the disease incidence and vaccine coverage in Japan is the same as in Tsu City, 18 770 children under 5 years old would be prevented from being hospitalized in Japan.

Similar to Tsu City, reduction in hospitalization due to rotavirus has been observed in the United States after introduction of RV5 into routine immunization in February 2006: by 31 December 2007, at least one dose of RV5 had been administered in 64% of children under 1 year old, and in 2008 to 2009, the hospitalization rate for rotavirus-coded diarrhoea declined by 60% from the baseline rates. (*16*) In Japan, rotavirus vaccination was optional in 2016. It is available based on self-pay, and vaccine history is not kept by local government. Based on our vaccine coverage study in Tsu City, the coverage rate was 56.5% for the first dose of rotavirus vaccine and 54.4% for the complete series. Even with those coverage rates, a decrease in the number of patients both in hospital as well as outpatient clinic settings is apparent.

Significant decreases were observed among children under 1 year old, between 1 and 2 years old and between 2 and 3 years old after the introduction of rotavirus vaccines. On the other hand, incidence did not change significantly among children in the 3 years old or above age group. Taking into consideration that the vaccine was introduced in late 2011 in Japan, the majority of children older than 3 years were probably not vaccinated with rotavirus vaccine. In the United States, herd immunity effect was seen after the vaccine coverage increased. (*17*, *18*) To obtain herd immunity effect from rotavirus vaccines in Japan, achieving higher vaccination coverage seems necessary and inclusion of the vaccine into the National Immunization Programme is one approach. Despite the significant reduction of hospitalization rates among children under 3 years of age, the hospitalization rate is still higher among children aged between 1 and 3 years compared to older children. This emphasizes the need to increase vaccination coverage in young children.

Rotavirus genotype G1 was the dominant type in Japan from the late 1980s to 2000. After that, G1 temporarily decreased and G3 became dominant. However G1 re-emerged and G3 decreased in 2004–2005. (*19*) In Japan, the majority of rotavirus vaccines at this time are RV1 which contains one strain of live attenuated human rotavirus genotype G1P[8]. We analysed rotavirus genotypes from the stool sample collected in this study. The proportions of circulating genotypes between hospitalizations and outpatient visits were very similar. During our study period, the main circulating genotypes shifted from G3P[8] to G1P[8] in 2011–2012 to G2P[4] in 2013–2014 and then back to G1P[8] in 2014–2015, although only a few cases were identified in 2013–2015. Recent reports from Belgium, Brazil, Republic of Korea, Nicaragua and the United States showed that the percentage of rotavirus disease due to type G2P[4] rotavirus increased after vaccine introduction. (*16*, *20*-*24*) However, the increase of G2P[4] was temporary in countries such as Brazil and Nicaragua, which is similar to what we observed in Tsu City. A study in 11 Latin American countries and Finland reported that the efficacy of RV1 against severe rotavirus gastroenteritis caused by type G1P[8] strains was 90.8% (*P* < 0.001) and against strains sharing only the P[8] antigen (G3P[8], G4P[8] and G9P[8]) was 87.3% (*P* < 0.001); efficacy against the fully heterotypic G2P[4] strains was 41.0% (*P* = 0.30). (*25*) Another study in six European countries also reported lower efficacy of RV1 against any rotavirus gastroenteritis caused by the G2 type (58.3%) compared to other G types, although the efficacy against severe rotavirus gastroenteritis caused by the G2 type was as high as that for other G types (85.5%). (*26*) However, a study in the United States reported high efficacy (94%) of RV1 against G2P[4] disease. (*27*) Thus, it is difficult to conclude that the serotype shift we observed may be a representation of selective pressure of vaccine or decreased vaccine effectiveness over time or both. The finding could also be an artefact of small numbers. Since we continue our study at the same site, continuous monitoring of the genotype is important.

Our study has some limitations. First, we were unable to collect stool samples from all hospitalized acute gastroenteritis cases. We did not use enema and rectal swab to collect samples, and some patients who didn’t provide stool specimens might have been missed, although we think the numbers are few. Second, this study is confined to just one city in Japan and is not nationally representative. Third, because rotavirus vaccine is optional in Japan at this time, it is difficult to assess accurate vaccine coverage in an area. However, very high attendance at 18-month check-ups in our area means that our estimated coverage rate should be relatively close to the actual coverage rate. Finally, this study is based on surveillance data of rotavirus gastroenteritis, and it is not a study to assess causality between vaccination and reduction in disease. There may have been unmeasured changes occurring during the study period which contributed to the decline in rotavirus gastroenteritis.

## Conclusions

In summary, after the introduction of rotavirus vaccine in Japan in 2011, we observed a reduction in the incidence of rotavirus gastroenteritis hospitalizations and outpatient visits in Tsu City among children. To maximize the impact of vaccination and achieve herd immunity, we recommend including the rotavirus vaccine in the National Immunization Programme in Japan as a mean to improve vaccine coverage. Continued testing for genotypes is important in monitoring possible vaccine-induced selective pressure and informing use of vaccines.
